# Metagenomic Survey of Viral Diversity Obtained from Feces of Subantarctic and South American Fur Seals

**DOI:** 10.1371/journal.pone.0151921

**Published:** 2016-03-17

**Authors:** Mariana Kluge, Fabrício Souza Campos, Maurício Tavares, Derek Blaese de Amorim, Fernanda Pedone Valdez, Adriana Giongo, Paulo Michel Roehe, Ana Claudia Franco

**Affiliations:** 1 Virology Laboratory, Department of Microbiology, Immunology and Parasitology, Institute of Basic Health Sciences, UFRGS (Federal University of Rio Grande do Sul), Porto Alegre, Rio Grande do Sul, Brazil; 2 CECLIMAR (Center for Coastal, Limnology and Marine Studies), UFRGS (Federal University of Rio Grande do Sul), Imbé, Rio Grande do Sul, Brazil; 3 Genomic and Molecular Biology Laboratory, PUCRS (Pontifical Catholic University of Rio Grande do Sul), Porto Alegre, Rio Grande do Sul, Brazil; 4 IPR (Institute of Petroleum and Natural Resources), PUCRS (Pontifical Catholic University of Rio Grande do Sul), Porto Alegre, Rio Grande do Sul, Brazil; University of Minnesota, UNITED STATES

## Abstract

The Brazilian South coast seasonally hosts numerous marine species, observed particularly during winter months. Some animals, including fur seals, are found dead or debilitated along the shore and may harbor potential pathogens within their microbiota. In the present study, a metagenomic approach was performed to evaluate the viral diversity in feces of fur seals found deceased along the coast of the state of Rio Grande do Sul. The fecal virome of two fur seal species was characterized: the South American fur seal (*Arctocephalus australis*) and the Subantarctic fur seal (*Arctocephalus tropicalis*). Fecal samples from 10 specimens (*A*. *australis*, n = 5; *A*. *tropicalis*, n = 5) were collected and viral particles were purified, extracted and amplified with a random PCR. The products were sequenced through Ion Torrent and Illumina platforms and assembled reads were submitted to BLASTx searches. Both viromes were dominated by bacteriophages and included a number of potentially novel virus genomes. Sequences of picobirnaviruses, picornaviruses and a hepevirus-like were identified in *A*. *australis*. A rotavirus related to group C, a novel member of the *Sakobuvirus* and a sapovirus very similar to *California sea lion sapovirus 1* were found in *A*. *tropicalis*. Additionally, sequences of members of the *Anelloviridae* and *Parvoviridae* families were detected in both fur seal species. This is the first metagenomic study to screen the fecal virome of fur seals, contributing to a better understanding of the complexity of the viral community present in the intestinal microbiota of these animals.

## Introduction

Every year, hundreds of marine species arrive at the coast of Rio Grande do Sul, the southernmost state in Brazil. Among these species, which include birds, turtles and mammals, fur seals are regular visitors that can be observed near or on-shore. These animals are driven to this region by the Malvinas current, particularly during winter months [1–3]. Although some fur seals may reach the coast to rest, several are found dead or debilitated along the shore and the cause of their weakness or death cannot always be determined [4,5]. Few studies have attempted to identify the pathogens that infect these populations and their roles as etiological agents of diseases and as potential zoonotic agents, especially those concerned with viruses [6–10]. While the virome of marine mammals has already been investigated [11], these studies have been restricted to species native to the northern hemisphere. Little is known about the viruses that infect marine mammals limited to the southern hemisphere and the effects of this geographical difference on their virome profiles.

Here, we evaluated the viral diversity of two species of pinnipeds from the Otariidae family from the southern hemisphere: the South American fur seal (*Arctocephalus australis*) and the Subantarctic fur seal (*Arctocephalus tropicalis*). While the South American fur seal is found along the Pacific and Atlantic coast of South America, the Subantarctic fur seal has a broader range that extends from the South Atlantic to Indian ocean islands. The South American fur seal is more frequently sighted in Rio Grande do Sul coast, mostly juveniles, due to the proximity of its closest breeding colony, located in the neighboring country of Uruguay. By contrast, the closest Subantarctic fur seals colonies are located at more than 4,000 km away at the south Atlantic islands of Gough and Tristan da Cunha [3,12]. Juveniles and adults specimens of Subantarctic fur seals reach the Atlantic coast with the help of ocean currents, and it is known that juveniles do not stay in the colonies during breeding seasons, while adults can travel long distances after mating [1,13].

The aim of this study was to examine the fecal virome of two species of fur seals whose cadavers were found along the shore of Rio Grande do Sul state. Anelloviruses, parvoviruses and picornaviruses were identified, as well as potential new members of *Sakobuvirus*, *Picobirnavirus* and *Rotavirus*. A sapovirus very similar to *California sea lion sapovirus 1* was found in the Subantarctic fur seal, and a hepevirus-like sequence was identified. The data provides a preliminary characterization of the viruses that occur within fur seals populations of the southern hemisphere.

## Materials and Methods

### Sample Collection

**Fig 1:**
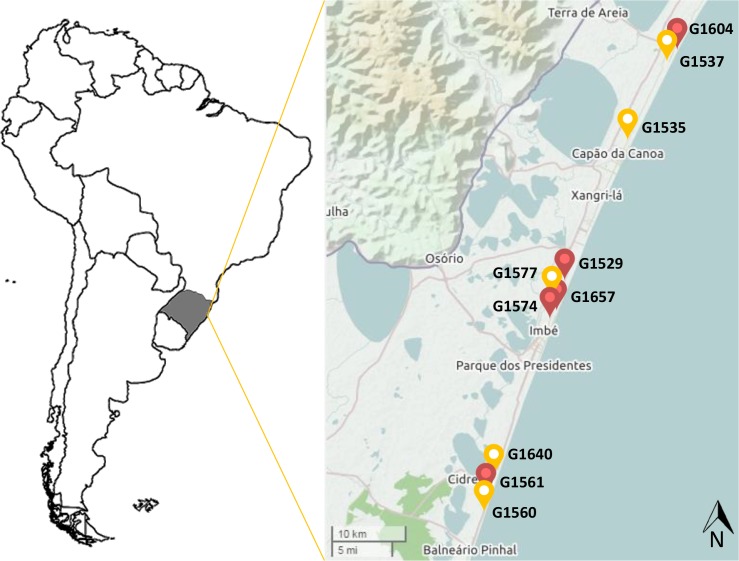
Sample location map. Map indicating the location of where the samples were collected along the coast of the State of Rio Grande do Sul, Brazil (shaded). The map was extracted from the Open Street Map[15] database.

Fecal samples from 10 specimens (*A*. *australis*, n = 5; *A*. *tropicalis*, n = 5) were collected directly from the intestines of deceased fur seals found along shores between August 2012 and September 2013 by the Center for Coastal, Limnology and Marine Studies (CECLIMAR) team. Samples for each species were pooled and kept at -80°C until processing. All samples from this study were collected in strict accordance with the Brazilian law, and the license for collecting zoological material was granted by SISBIO/Ministry of the Environment (License number: 20185–4). The location and information about the specimens are provided in Table 1 and Fig 1.

**Table 1:** Samples used in this study.

Pool no.	No.	Species	Length (cm)	Weight (kg)	Sex	Carcass classification code*	Date of collection (dd/mm/yyyy)	Collection location	Geolocation latitude/ longitude (decimal)
1	G1529	South American fur seal *Arctocephalus australis*	94.2	10.6	Male	2	02/08/2012	Osório, RS	-29.878119/-50.073224
1	G1560	South American fur seal *Arctocephalus australis*	92	9.5	Male	2	09/08/2012	Cidreira, RS	-30.165946/-50.197728
1	G1574	South American fur seal *Arctocephalus australis*	89.4	10.8	Male	2	16/08/2012	Imbé, RS	-29.94758/-50.105842
1	G1604	South American fur seal *Arctocephalus australis*	92	15.5	Male	2	31/08/2012	Capão da Canoa, RS	-29.657469/-49.954338
1	G1657	South American fur seal *Arctocephalus australis*	88	12	Male	2	11/09/2013	Imbé, RS	-29.94579/-50.10498
2	G1535	Subantarctic fur seal *Arctocephalus tropicalis*	91.9	8.4	Male	2	02/08/2012	Capão da Canoa, RS	-29.730323/-49.995557
2	G1537	Subantarctic fur seal *Arctocephalus tropicalis*	90.6	9	Male	2	02/08/2012	Capão da Canoa, RS	-29.665492/-49.959170
2	G1561	Subantarctic fur seal *Arctocephalus tropicalis*	91.9	8.8	Male	2	09/08/2012	Cidreira, RS	-30.174591/-50.200809
2	G1577	Subantarctic fur seal *Arctocephalus tropicalis*	80.5	7.1	Male	3	16/08/2012	Osório, RS	-29.917806/-50.091768
2	G1640	Subantarctic fur seal *Arctocephalus tropicalis*	157.5	40.9	Male	2	25/07/2013	Tramandaí, RS	-30.13273/-50.18535

*Code for carcass classification according to Geraci & Lounsbury (1993)[14]: freshly dead, edible **(2)**; and decomposed, but organs basically intact **(3)**.

### Viral Particle Purification and Nucleic Acid Extraction

Fecal samples were suspended in Hank's balanced salt solution, vigorously vortexed and then centrifuged at 2500 × g for 90 min at 4°C. The supernatant was again centrifuged for 10 min at maximum speed and then filtered through a 0.45 μm syringe filter (MF-Millipore). The viral particles were harvested and pelleted on a 25% sucrose cushion by ultracentrifugation at 190000 × g for 4h at 4°C. The pellet was resuspended in TE buffer and clarified by emulsifying with 1/1 (v/v) chloroform and centrifugation. In order to remove nucleic acids not protected by the capsid, the purified samples were treated with 100 U of DNase I (Roche) and 20 U of RNase (Invitrogen) at 37°C for 2h, as similar to other studies [16,17].

Viral genomes were extracted via commercial kits (PureLink® Viral RNA/DNA Invitrogen for DNA extraction; RNeasy® Mini Kit Qiagen for RNA) and processed as described by [18] with minor modifications. Briefly, a complementary strand of extracted DNA (5 μl) was synthesized using the Klenow fragment DNA polymerase (New England Biolabs) and primer K-randoms (GAC CAT CTA GCG ACC TCC AC*M NN MNM*) designed by [19]. For the extracted RNA (10 μl), a reverse transcription using the primer K-randoms was carried out prior the second strand synthesis using Klenow fragment DNA polymerase.

### Library Construction for Metagenomic Sequencing

A random PCR was performed in a final volume of 50 μL, containing 5 μL of template, 0.8 μM of the fixed portion of primer K-randoms (GAC CAT CTA GCG ACC TCC AC), 0.2 mM of each dNTP, 1X PCR Buffer, 2.5 mM MgCl_2_, and 1 U of *Taq* DNA polymerase. Amplification conditions consisted of an initial denaturation cycle at 95°C for 5 min, followed by 35 cycles for amplification (95°C for 1 min, 53°C for 1 min and 72°C for 1 min), and final extension at 72°C for 7 min. The products were visualized by 1% agarose gel electrophoresis, purified and processed for Ion Torrent (Life Technologies, USA) using a 316 Ion chip, which was performed by the Genomic and Molecular Biology Laboratory from the Pontifical Catholic University of Rio Grande do Sul. The same process, including the random PCR, was repeated for Illumina MiSeq platform sequencing using Kit v2 in the 300-cycles (2x150) format performed by Fepagro Animal Health Institute of Veterinary Research Desidério Finamor (IPVDF), Eldorado do Sul, Brazil.

### Bioinformatics

Ion Torrent reads were trimmed using PRINSEQ (prinseq.sourceforge.net) and the quality of the sequences was analyzed with FastQC (www.bioinformatics.babraham.ac.uk/projects/fastqc/). Trimmed reads were assembled *de novo* by using MetaVelvet v1.2.01 (metavelvet.dna.bio.keio.ac.jp) with a k-mer of 51. Illumina reads were trimmed for primers using Geneious 8.1.3 and *de novo* assembled with St. Petersburg genome assembler (SPAdes) 3.5.0 (bioinf.spbau.ru/spades). The resulting contigs (>100bp) were submitted to BLASTx search against the National Center for Biotechnology Information (NCBI, www.ncbi.nlm.nih.gov) non-redundant database (nr) and its viral database by using an E-value cutoff of 1e-05. The contigs were classified into eukaryotic viruses, bacteriophages, bacterium, eukaryotes and unknown based on lowest E-value. Contigs of eukaryotic viruses were used for sequence and phylogenetic analyses and bacteriophage sequences were not further analyzed. The GenBank accession numbers for the sequences derived in this study are: KR261062, KR261063, KR261065, KR816222, KR816223 (fur seal anelloviruses); KR261066-KR261068, KR261070-KR261075, KR261077-KR261079, KR816217, KR816218, KR816220, KR816221 (fur seal parvoviruses); KR106199-KR106202, KR816213, KR816215, KR337994 (fur seal picornaviruses); KR072975-KR072979, KR072981, KR072982, KR072984 (fur seal sakobuvirus); KR106194-KR106196, KR106198, KR816216 (fur seal picobirnavirus); KR072985-KR072990 (fur seal rotavirus), KR827461 (fur seal hepevirus); KR072992, KR072994, KR072995 (fur seal sapovirus). The sequence data obtained from this study is available at the NIH Sequence Read Archive (SRA) under the study accession number SRP070196.

### Phylogenetic Analysis

Nucleotide or translated amino acid sequences from the contigs of anellovirus, parvovirus, picornavirus, picobirnavirus, rotavirus, sapovirus and hepevirus-like were aligned with MUSCLE (www.drive5.com/muscle) and phylogenetic trees were built using MEGA6 [20]. Trees were constructed by the neighbor-joining (NJ) method [21] with a bootstrap of 1000 replicates, p-distance model, and gaps were treated as pairwise deletion. The contig sequences from this study were compared with other selected gene sequences available in the GenBank.

## Results

### Overview

A substantial proportion of the assembled reads detected in both fur seals species have no significant similarity to any of the sequences deposited to date at GenBank. About 70% of the assembled reads from the Ion Torrent platform had no significant hits, whereas in Illumina NGS apparatus the sequences with no identified matches reached 35% (cutoff for significant as <1e-05 BLASTx E score). The same divergence was observed with the number of bacterial hits, however, Ion Torrent had the lowest number of hits (about 25%) when compared to Illumina (about 60%).

**Fig 2:**
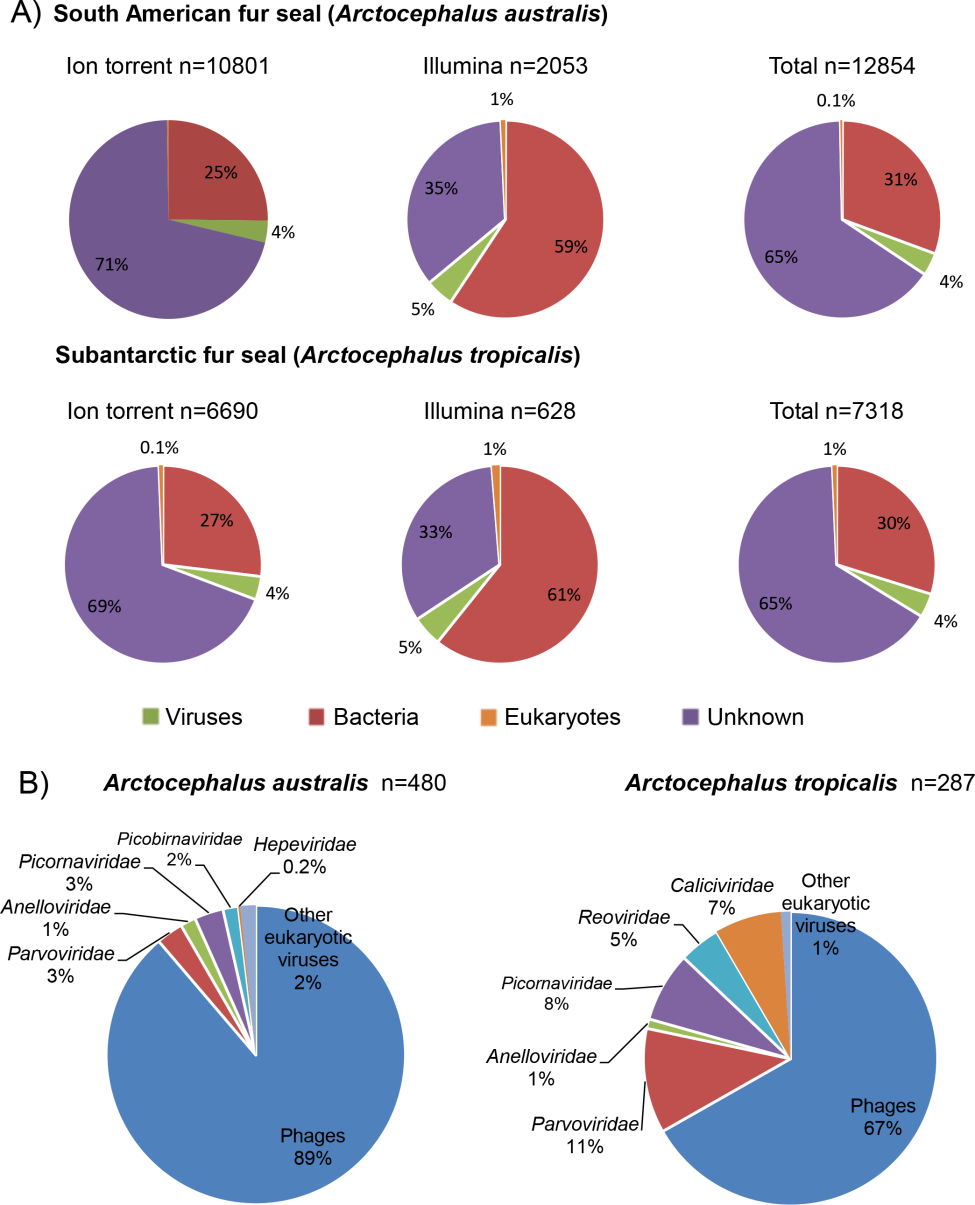
Taxonomic classification of assembled reads (>100bp). (A) Pie charts of assembled reads based on BLASTx best E-scores (cutoff: 10e-05) against the GenBank non-redundant and viral databases. (B) Taxonomic distribution of viruses for each fur seal species.

The viral component detected in either of the sequencing platforms represented 4–5% of total sequences, regardless of the fur seal species analyzed. Most of the viral hits were from bacteriophages, in agreement with previous studies of bats and dromedary fecal viromes [22–24]. Some of the contigs from eukaryotic viruses displayed low similarity to currently known viruses and, as such, may represent novel viruses. The Subantarctic fur seal was found to carry a larger proportion of identifiable sequences of eukaryotic viruses (95 hits, corresponding to 33% of total assembled reads assigned to viruses) when compared to the South American fur seal (53 hits, corresponding to 11% of total assembled reads assigned to viruses). The proportional taxonomic composition of the assembled reads is shown in Fig 2.

### South American Fur Seal (*Arctocephalus australis*)

Ion Torrent sequencing generated a total of 475,511 reads with an average length of 191 bp which were trimmed to a final number of 282,732 reads. MetaVelvet *de novo* assembly of trimmed reads resulted in 10,801 contigs (>100 bp). Illumina sequencing generated a total of 496,016 paired-end reads (average length of 149 bp) which were trimmed for primers and assembled *de novo* with St. Petersburg genome assembler (SPAdes) into 2,053 contigs (>100 bp). BLASTx results from the Ion Torrent contigs revealed sequences with similarity to the eukaryotic virus families *Parvoviridae* (11 contigs), *Anelloviridae* (5), *Picornaviridae* (10), *Picobirnaviridae* (5) and invertebrate virus (1). Illumina contigs displayed similarity to genomes of members of the families *Parvoviridae* (3), *Anelloviridae* (3), *Picornaviridae* (5), *Picobirnaviridae* (3) and *Hepeviridae* (1), among other viruses that infect fish, small invertebrates and insects (7). Contigs with significant BLASTx hits and their GenBank accession numbers are shown in Table 2.

**Table 2:** Contigs (>200bp) with significant BLASTx hits to known eukaryotic viruses obtained from the South American fur seals (*Arctocephalus australis*).

Contig ID	Accession number	Length (nt)	Family/Genus	Genome	Product	Best hit	Amino acid identity (%)	E-value
58	KR261062	1292	*Anelloviridae*	ssDNA	putative ORF1	ORF1 [Seal anellovirus 5] (KM262782)	35	5e-61
59	KR261063	480	*Anelloviridae*	ssDNA	putative ORF1	ORF1 [Seal anellovirus 5] (KM262782)	45	1e-14
62	KR816222	1080	*Anelloviridae*	ssDNA	putative ORF1	ORF1 [Torque teno sus virus 1a] (HM633252)	84	0.0
53	KR261066	616	*Parvoviridae*	ssDNA	capsid protein	VP2 [Tusavirus 1] (KJ495710)	46	8e-46
54	KR261067	334	*Parvoviridae*	ssDNA	capsid protein	capsid protein [Canine parvovirus 2a](HM042734)	50	2e-29
55	KR261068	460	*Parvoviridae*	ssDNA	NS1	NS1 [Solwezi bufavirus] (LC011438)	43	1e-23
57	KR261070	237	*Parvoviridae*	ssDNA	capsid protein	VP2 [Fox parvovirus] (KC692368)	46	3e-11
63	KR816220	344	*Parvoviridae*	ssDNA	NS1	NS1 [Tusavirus 1] KJ495710)	82	1e-65
34	KR106199	561	*Picornaviridae*	+ssRNA	polyprotein	polyprotein [Hepatitis A virus] (FJ360731)	36	3e-15
35	KR106200	707	*Picornaviridae*	+ssRNA	polyprotein	capsid protein [Hepatitis A virus] (AF365952)	37	1e-39
36	KR106201	519	*Picornaviridae*	+ssRNA	polyprotein	putative 3C [Avian encephalomyelitis virus] (NP_653151)	39	2e-26
37	KR106202	285	*Picornaviridae*	+ssRNA	polyprotein	polyprotein [Bat picornavirus] (KJ641684)	38	5e-12
65	KR816213	466	*Picornaviridae*	+ssRNA	polyprotein	1B VP2 mature peptide [Hepatitis A virus] (NP_041008)	52	2e-50
67	KR816215	318	*Picornaviridae*	+ssRNA	polyprotein	hypothetical protein [Avian encephalomyelitis virus] (AJ006950)	32	2e-09
29	KR106194	217	*Picobirnaviridae Picobirnavirus*	dsRNA	RNA-dependent RNA polymerase	RNA dependent RNA polymerase [Human picobirnavirus] (AB517735)	52	3e-13
30	KR106195	968	*Picobirnaviridae Picobirnavirus*	dsRNA	RNA-dependent RNA polymerase	RNA-dependent RNA polymerase [Fox picobirnavirus] (KC692366)	71	1e-169
31	KR106196	240	*Picobirnaviridae Picobirnavirus*	dsRNA	RNA-dependent RNA polymerase	putative RNA-dependent RNA polymerase [Dromedary picobirnavirus] (KM573806)	77	3e-34
33	KR106198	293	*Picobirnaviridae Picobirnavirus*	dsRNA	capsid protein	hypothetical protein [Human picobirnavirus] (GU968923)	35	1e-08
64	KR816216	330	*Picobirnaviridae Picobirnavirus*	dsRNA	RNA-dependent RNA polymerase	putative RNA-dependent RNA polymerase [Dromedary picobirnavirus] (KM573806)	82	3e-61
73	KR827461	661	*Hepevirus-like*	+ssRNA	polyprotein	nonstructural protein [Hepatitis E virus] (JQ026407)	27	4e-07

### Subantarctic Fur Seal (*Arctocephalus tropicalis*)

Ion Torrent sequencing generated a total of 784,917 reads with an average length of 184 bp which were trimmed into 288,611 reads. Trimmed reads were *de novo* assembled with MetaVelvet into 6,690 contigs (>100 bp). Illumina sequencing generated a total of 1,253,988 paired-end reads (average length of 144 bp) which were trimmed for primers and *de novo* assembled with SPAdes into 628 contigs (>100 bp). For Ion Torrent contigs, the eukaryotic virus families with significant similarity to results from BLASTx searches were *Parvoviridae* (24 contigs), *Anelloviridae* (2), *Picornaviridae* (19), *Reoviridae* (13), *Caliciviridae* (18), other insect viruses (2) and a circovirus-like hit (1). Illumina contigs had similarity with *Parvoviridae* (9), *Anelloviridae* (1), *Picornaviridae* (3), *Caliciviridae* (3) and *Reoviridae* (1). Contigs with significant BLASTx hits and their GenBank accession numbers are shown in Table 3.

**Table 3:** Contigs (>200bp) with significant BLASTx hits to known eukaryotic viruses obtained from the Subantarctic fur seals (Arctocephalus tropicalis).

Contig ID	Accession number	Length (nt)	Family/Genus	Genome	Product	Best hit	Amino acid identity (%)	E-value
52	KR261065	347	*Anelloviridae*	ssDNA	putative ORF2	ORF2 [Torque teno zalophus virus 1] (NC_012126)	78	5e-18
72	KR816223	467	*Anelloviridae*	ssDNA	putative ORF2 and ORF1	ORF1 [Torque teno sus virus 1a] (HM633252)	88	4e-39
40	KR261071	1519	*Parvoviridae*	ssDNA	capsid protein	VP2 [Tusavirus 1] (KJ495710)	39	1e-85
41	KR261072	1648	*Parvoviridae*	ssDNA	NS1	NS1 [Miniopterus schreibersii parvovirus] (KC154061)	57	7e-131
42	KR261073	628	*Parvoviridae*	ssDNA	NS1	nonstructural protein NS1 [Tumor virus X] (KJ631100)	44	2e-43
43	KR261074	565	*Parvoviridae*	ssDNA	NS1	NS1 [Turkey parvovirus TP1-2012/HUN] (KF925531)	36	2e-13
44	KR261075	612	*Parvoviridae*	ssDNA	capsid protein	putative VP1 [Tusavirus 1] (KJ495710)	39	2e-18
46	KR261077	349	*Parvoviridae*	ssDNA	capsid protein	VP protein [Canine parvovirus] (KM235293)	55	2e-26
47	KR261078	957	*Parvoviridae*	ssDNA	NS1	non-structural protein 1 [Chipmunk parvovirus] (U86868)	37	1e-26
48	KR261079	301	*Parvoviridae*	ssDNA	capsid protein	capsid protein [Canine parvovirus 2b] (JQ730016)	53	6e-25
68	KR816217	438	*Parvoviridae*	ssDNA	capsid protein	putative VP1 [Tusavirus 1] (KJ495710)	42	1e-18
69	KR816218	322	*Parvoviridae*	ssDNA	capsid protein	capsid protein VP2 [Mpulungu bufavirus] (NC_026815)	36	1e-07
70	KR816221	319	*Parvoviridae*	ssDNA	NS1	NS1 [Miniopterus schreibersii parvovirus] (KC154061)	41	1e-14
61	KR337994	438	*Picornaviridae*	+ssRNA	polyprotein	AEV polyprotein [Avian encephalomyelitis virus] (NC_003990)	34	4e-15
12	KR072975	1271	*Picornaviridae Sakobuvirus*	+ssRNA	polyprotein	polyprotein [Feline sakobuvirus A] (NC_022802)	58	6e-126
13	KR072976	477	*Picornaviridae Sakobuvirus*	+ssRNA	polyprotein	polyprotein [Kobuvirus SZAL6-KoV/2011/HUN] (KJ934637)	52	4e-12
14	KR072977	289	*Picornaviridae Sakobuvirus*	+ssRNA	polyprotein	polyprotein [Feline sakobuvirus A] (NC_022802)	66	3e-22
15	KR072978	273	*Picornaviridae Sakobuvirus*	+ssRNA	polyprotein	VP3 [Feline sakobuvirus A] (YP_008802588)	66	1e-34
16	KR072979	227	*Picornaviridae Sakobuvirus*	+ssRNA	polyprotein	VP1 [Feline sakobuvirus A] (YP_008802588)	59	2e-12
18	KR072981	466	*Picornaviridae Sakobuvirus*	+ssRNA	polyprotein	2C [Feline sakobuvirus A] (YP_008802588)	59	3e-58
20	KR072982	767	*Picornaviridae Sakobuvirus*	+ssRNA	polyprotein	3D [Feline sakobuvirus A] (YP_008802588)	65	3e-117
22	KR072984	430	*Picornaviridae Sakobuvirus*	+ ssRNA	polyprotein	3D [Feline sakobuvirus A] (YP_008802588)	73	1e-67
23	KR072985	469	*Reoviridae Rotavirus*	dsRNA	NSP2	nonstructural protein 2 [Bovine rotavirus C] (AB874653)	69	3e-66
24	KR072986	412	*Reoviridae Rotavirus*	dsRNA	NSP3	nonstructural protein 3 [Bovine rotavirus C] (AB874654)	45	6e-33
25	KR072987	928	*Reoviridae Rotavirus*	dsRNA	VP1	VP1 [Bovine rotavirus C] (AB738412)	69	2e-137
26	KR072988	442	*Reoviridae Rotavirus*	dsRNA	VP3	VP3 [Human rotavirus C] (HQ185645)	51	5e-41
27	KR072989	357	*Reoviridae Rotavirus*	dsRNA	VP3	viral protein 3 [Bovine rotavirus C] (AB874621)	65	6e-46
28	KR072990	360	*Reoviridae Rotavirus*	dsRNA	VP7	outer capsid protein VP7 [Human rotavirus C] (JQ177070)	59	9e-42
02	KR072992	2932	*Caliciviridae Sapovirus*	+ ssRNA	polyprotein	polyprotein [California sea lion sapovirus 1] (JN420370)	98	0.0
06	KR072994	1400	*Caliciviridae Sapovirus*	+ ssRNA	polyprotein	polyprotein [California sea lion sapovirus 1] (JN420370)	98	0.0
07	KR072995	2408	*Caliciviridae Sapovirus*	+ ssRNA	polyprotein and VP2	polyprotein [California sea lion sapovirus 1] (JN420370)	96	0.0

### Anellovirus

**Fig 3:**
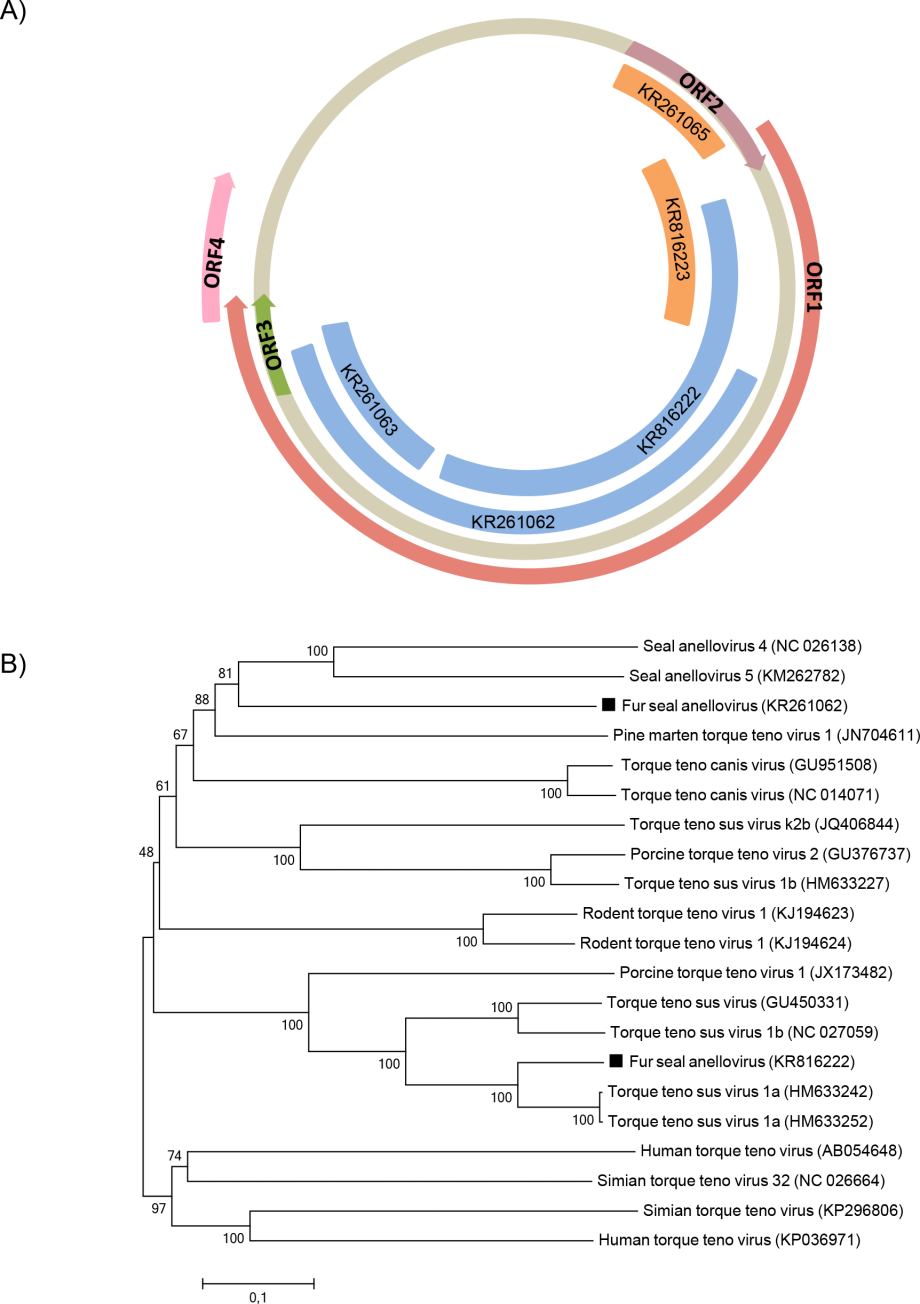
Phylogenetic analysis of fur seal anellovirus. (A) Schematic representation of the genome of anelloviruses using as example the torque teno virus (~3.8 kb). The blue bars represent the contigs from South American fur seal and the orange bars represent the contigs from Subantarctic fur seal. (B) Neighbor-joining phylogenetic tree based on the alignment of partial amino acid sequences (233 aa) from the ORF1 of 21 anelloviruses. Human and simian torque teno viruses were used as outgroup. The anellovirus sequences from South American fur seal identified in this study are labeled with black squares. The GenBank accession numbers of the viral sequences are shown in parentheses.

Anelloviruses are small, non-enveloped, circular ssDNA viruses belonging to the *Anelloviridae* family [25]. Anellovirus genome sequences were detected in both fur seal species (Fig 3A). Few sequences from the South American fur seal had the closest similarity to *Seal anellovirus 5*, with an amino acid identity ranging from 35–45% and covering up to 65% of ORF1. Another sequence from the Subantarctic fur seal had the closest similarity to *Torque teno zalophus virus1* (TTZV), with 78% amino acid identity and covering 69% of ORF2 of TTZV. Both fur seal species had sequences with the closest similarity to *Torque teno sus virus 1a*, with an amino acid identity ranging from 84–88% and coverage of up to 56% of ORF1.

Due to the high divergence within anelloviruses, ORF1 sequences are the most indicative to phylogenetic analyses [25]. Phylogenetic trees of partial ORF1 amino acid sequences obtained from South American fur seals showed that distinct anelloviruses grouped in different clusters: one most closely related to seal anelloviruses, whereas the other sequence was placed on the same clade as swine torque teno viruses (Fig 3B).

### Parvovirus

**Fig 4:**
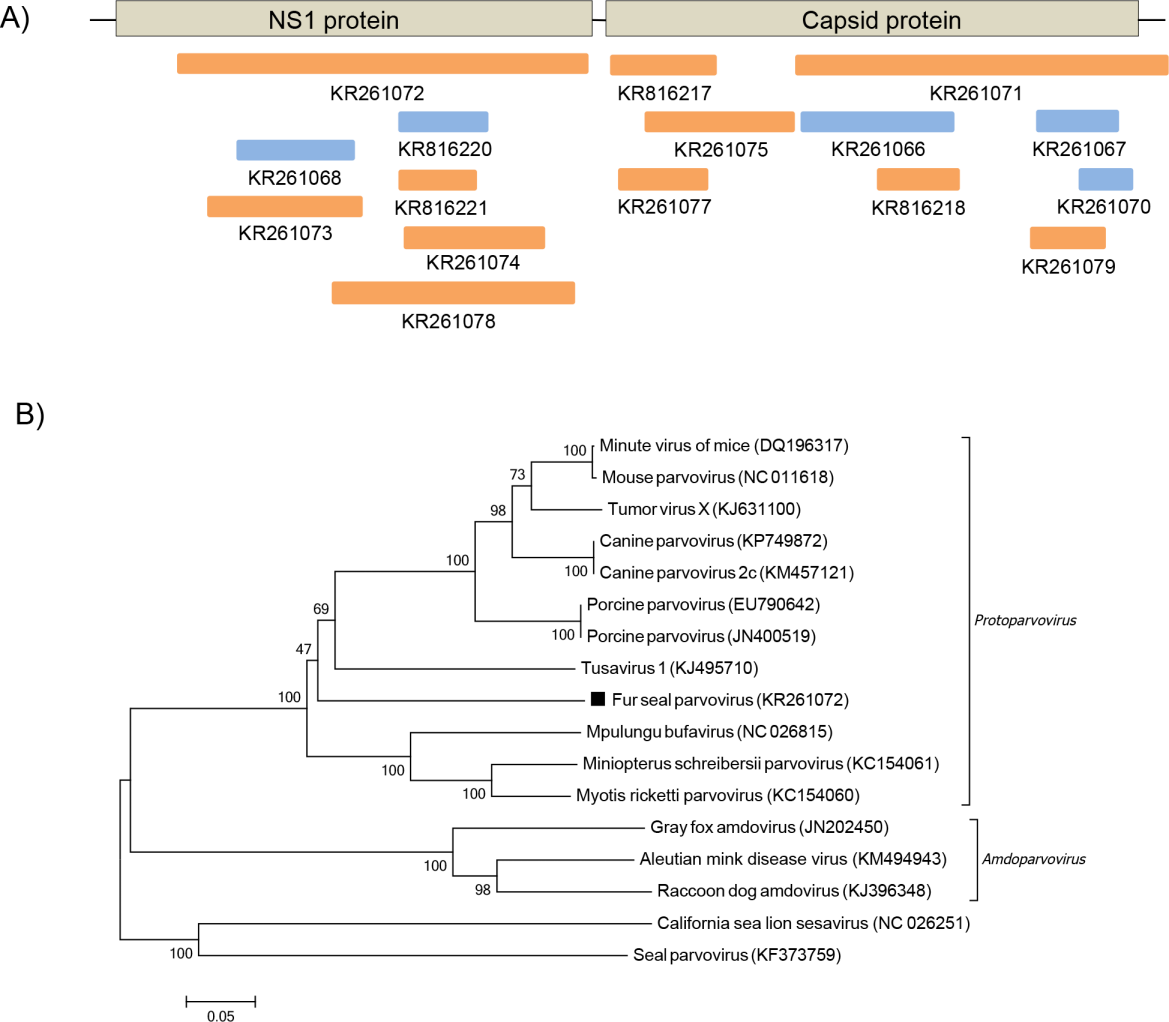
Phylogenetic analysis of fur seal parvovirus. (A) Schematic representation of the genome of parvoviruses using as example the tusavirus (~4.4 kb). The blue bars represent the contigs from South American fur seal and the orange bars represent the contigs from Subantarctic fur seal. (B) Neighbor-joining phylogenetic tree based on the alignment of partial amino acid sequences (261 aa) from the NS1 protein of 17 parvoviruses. *Seal parvovirus* and *California sea lion sesavirus* were used as outgroup. The parvovirus sequence from Subantarctic fur seal identified in this study is labeled with a black square. The GenBank accession numbers of the viral sequences are shown in parentheses.

Parvoviruses are non-enveloped linear ssDNA viruses, members of the *Parvoviridae* family. In this study, both fur seals species had sequences (Fig 4A) more closely related to mammal parvoviruses of the *Parvovirinae*, a subfamily that infects vertebrates and is currently divided into eight genera [26,27]. The amino acid identity of those sequences with members of the *Parvovirinae* ranged from 36–82%. Phylogenetic analysis of partial NS1 sequence, conserved within parvoviruses, showed that the fur seal parvovirus clustered with members of the *Protoparvovirus* genus, distant from previously described pinniped parvorviruses (Fig 4B).

### Picornavirus

**Fig 5:**
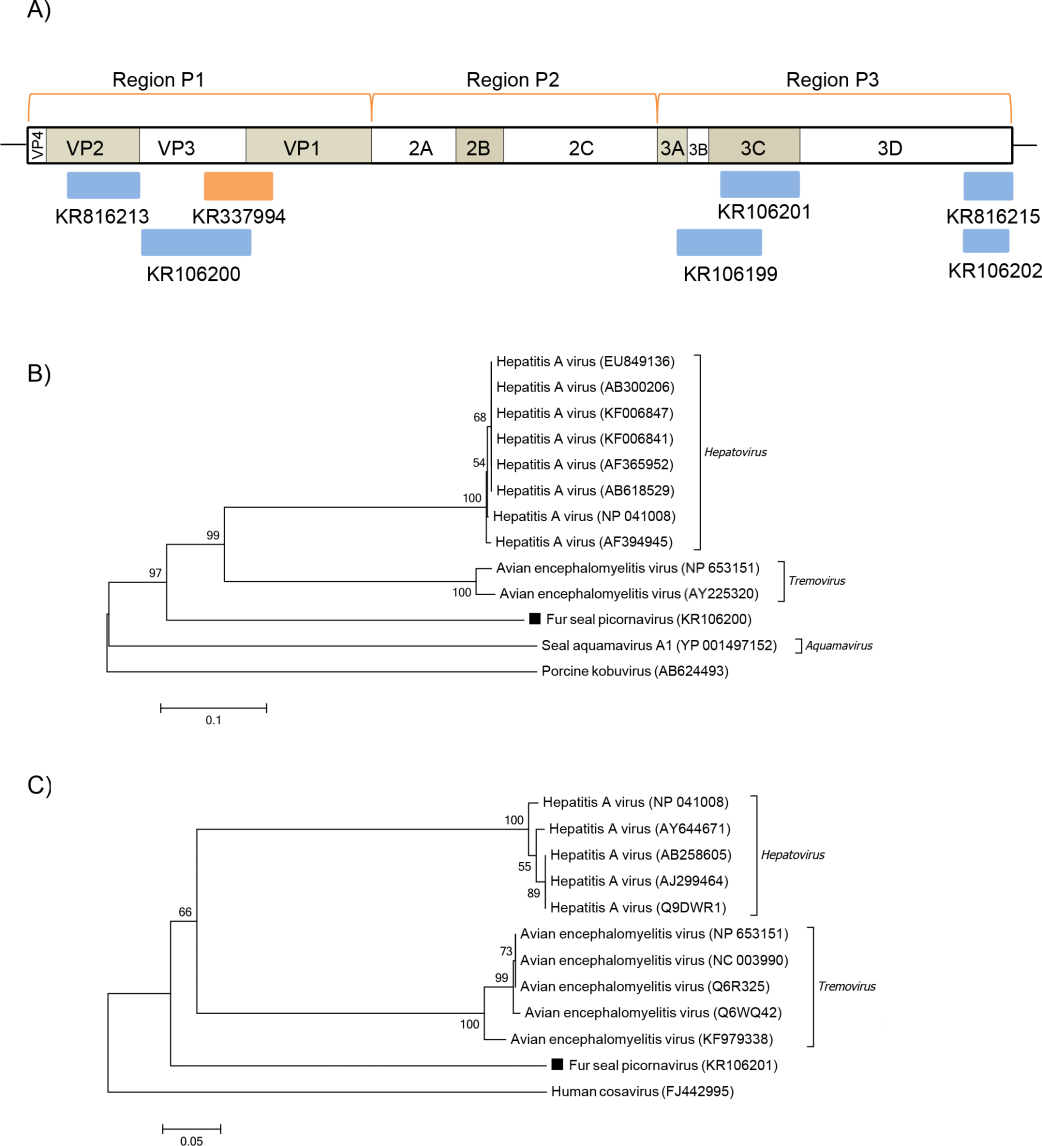
Phylogenetic analysis of fur seal picornavirus. (A) Schematic representation of the genome of picornaviruses using as an example the hepatits A virus (~7.4 kb). The blue bars represent the contigs from South American fur seal and the orange bars represent the contigs from Subantarctic fur seal. (B) Neighbor-joining phylogenetic tree based on the alignment of partial amino acid sequences (219 aa) from the P1 region of the polyprotein of 13 picornaviruses. *Porcine kobuvirus* was used as outgroup. (C) Neighbor-joining phylogenetic tree based on the alignment of partial amino acid sequences (122 aa) from the P3 region of the polyprotein of 12 picornaviruses. *Human cosavirus* was used as outgroup. The picornavirus sequences from South American fur seal identified in this study are labeled with a black square. The GenBank accession numbers of the viral sequences are shown in parentheses.

Picornaviruses are small, non-enveloped, positive sense ssRNA viruses of the *Picornaviridae* family, which has, to date, 29 recognized genera, though often increasing [28–30]. Picornavirus sequences more related to Hepatitis A (HAV) and Avian encephalomyelitis viruses (AEV) were detected in both fur seal species examined (Fig 5A). HAV belongs to *Hepatovirus* and AEV to *Tremovirus*, which are closely related genera [31]. The polyprotein sequences obtained here displayed between 32–39% of amino acid identity to both *Hepatovirus* and *Tremovirus* members, and one sequence shared 52% amino acid identity with HAV VP2. Phylogenetic analyses were based on the picornavirus polyprotein functional regions: P1, which encodes for structural proteins, and P2-P3, which encode for proteins involved in replication [28]. The analysis of partial P1 sequences identified here showed the fur seal picornavirus forming a monophyletic group with the *Hepatovirus* and *Tremovirus* genera, but on a different branch (Fig 5B). When partial sequences of P3 region were analyzed they still shared the same root, with the fur seal picornavirus placed in a similar way (Fig 5C).

**Fig 6:**
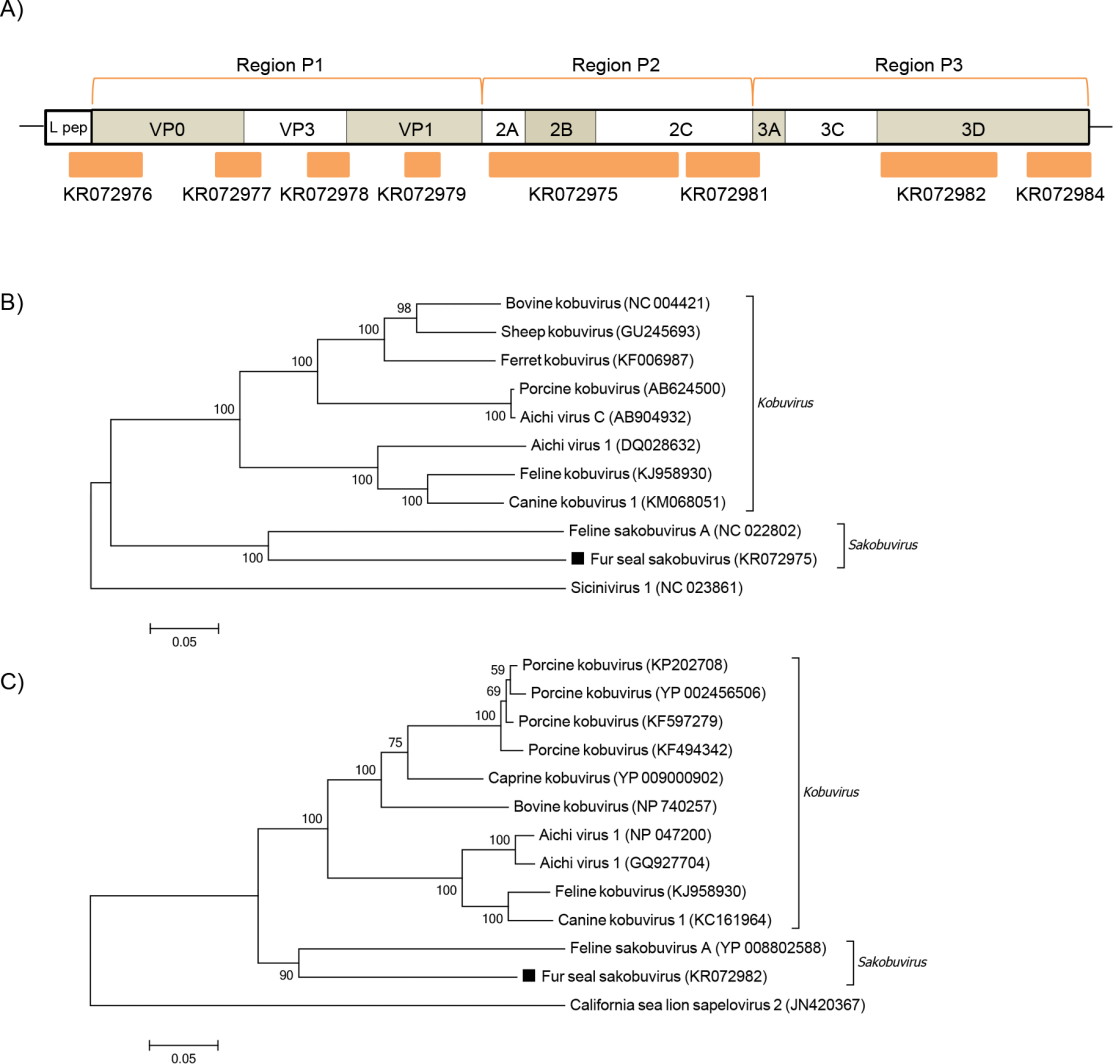
Phylogenetic analysis of fur seal sakobuvirus. (A) Schematic representation of the sakobuvirus genome using *Feline sakobuvirus A* (~7.8 kb—NC_022802) as a reference. The orange bars represent the contigs from Subantarctic fur seal. (B) Neighbor-joining phylogenetic tree based on the alignment of partial amino acid sequences (409 aa) from the P2 region of the polyprotein of 11 picornaviruses. *Sicinivirus 1* was used as outgroup. (C) Neighbor-joining phylogenetic tree based on partial amino acid sequences (255 aa) from the 3D region of the polyprotein of 13 picornaviruses. *California sea lion sapelovirus 2* was used as outgroup. The sakobuvirus sequences from the Subantarctic fur seal from this study used in phylogenetic analyses are labeled with a black square. The GenBank accession numbers of the viral sequences are shown in parentheses.

In addition to the above-mentioned picornaviruses, distinct members of this family were found only in Subantarctic fur seals. With the exception of one sequence whose best BLASTx hit had 52% of amino acid identity to a kobuvirus, all other contigs displayed the highest amino acid identity to *Feline sakobuvirus A* (FSVA), which ranged from 58–73% with a total coverage of 59% of its polyprotein (Fig 6A). One sequence displayed 59% of amino acid identity to FSVA VP1, and the amino acid identity of VP3 was of 66%. The sequence covering the 2C region had 59% of amino acid identity, while the two 3D sequences here identified ranged from 65–73% of FSVA 3D region. Phylogenetic analyses of partial P2 and P3 regions (Fig 6B and 6C, respectively) showed the fur seal picornavirus, temporarily named Fur seal sakobuvirus (FSSV), clustered with FSVA, member of the *Sakobuvirus* genus.

### Picobirnavirus

**Fig 7:**
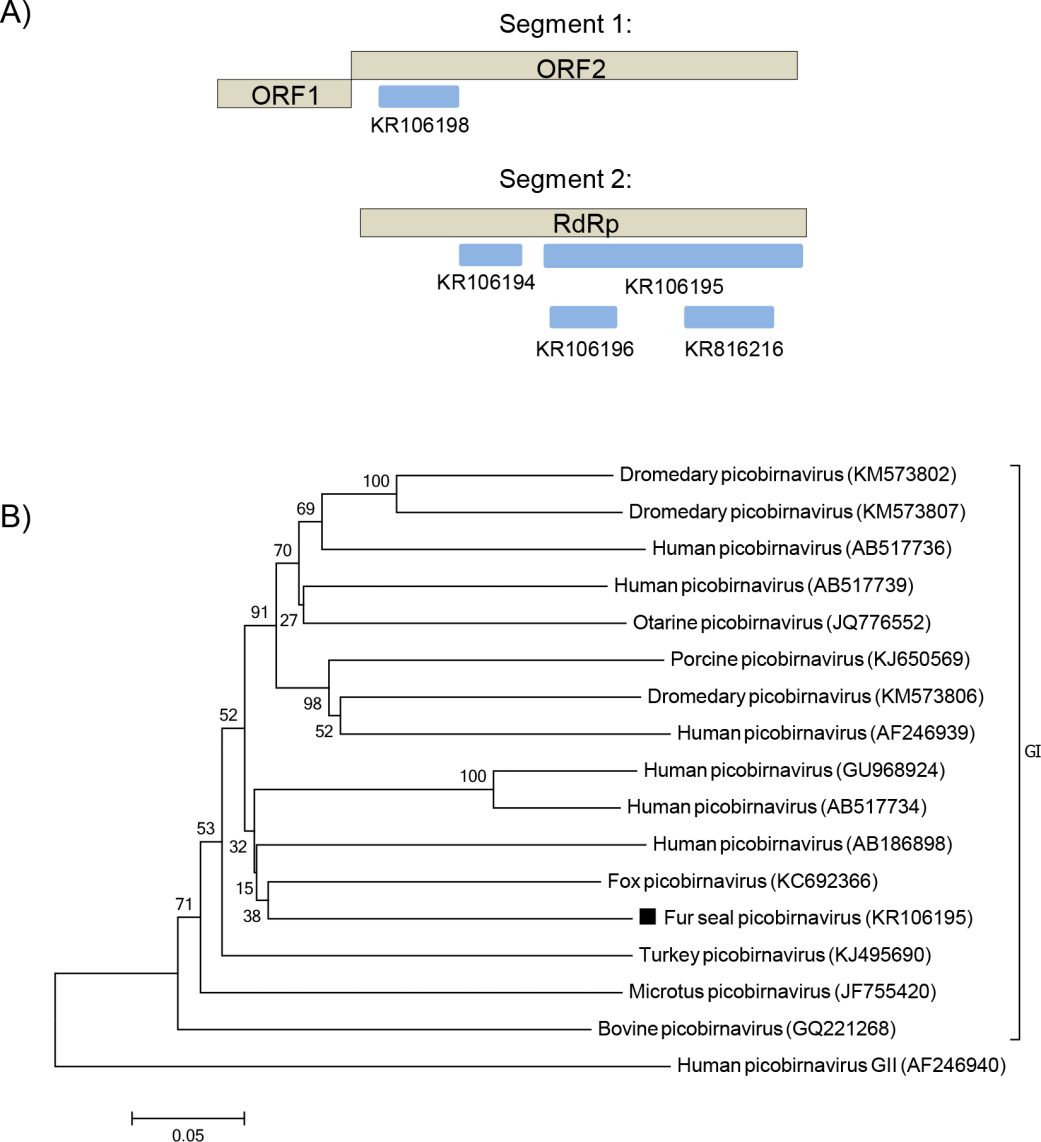
Phylogenetic analysis of fur seal picobirnavirus. (A) Schematic representation of the genome of picobirnaviruses using as an example the human picobirnavirus (~4.2 kb). The blue bars represent the contigs from South American fur seal. (B) Neighbor-joining phylogenetic tree based on the alignment of partial nucleotide sequences (743 bp) from the RdRp gene of 17 picobirnaviruses. Human picobirnavirus GII was used as outgroup. The picobirnavirus sequence from South American fur seal identified in this study is labeled with a black square. The GenBank accession numbers of the viral sequences are shown in parentheses.

Picobirnaviruses are small, non-enveloped, bisegmented dsRNA viruses of the *Picobirnaviridae* family. These highly variable viruses are classified in a sole genus, *Picobirnavirus*, which on its turn is divided into two genogroups (I and II), based on sequence similarities of the RNA-dependent-RNA-polymerase gene (RdRp) [32,33]. Sequences of picobirnavirus RdRp and capsid protein were detected in the South American fur seal samples (Fig 7A), having the highest similarity with members of genogroup I, with an amino acid identity ranging from 35–82%. Phylogenetic analyses of the partial RdRp gene (743 bp, which corresponds to 44% of the RdRp gene) confirmed that the fur seal picobirnavirus identified here clustered with members of genogroup I, with a nucleotide identity ranging from 60–68% (Fig 7B).

### Rotavirus

**Fig 8:**
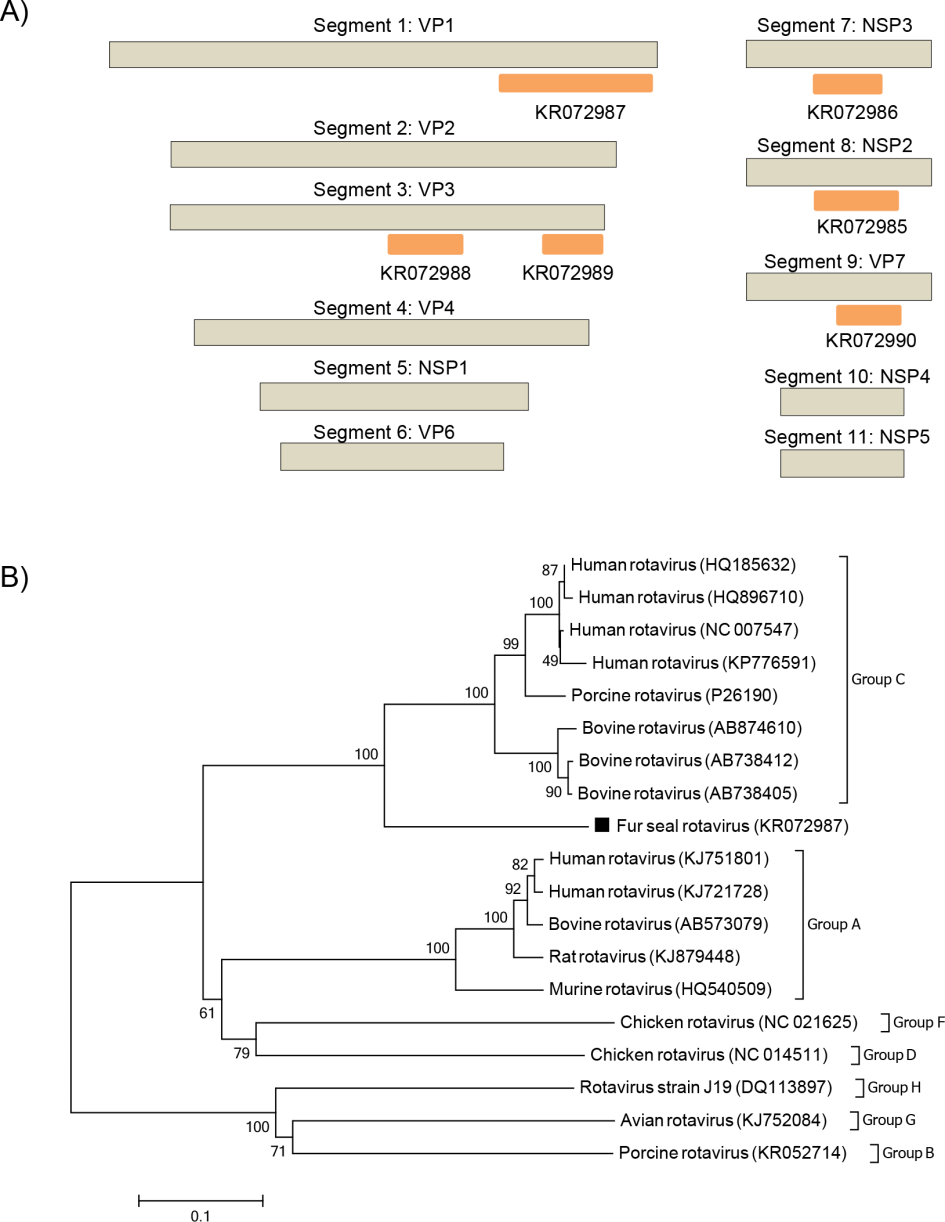
Phylogenetic analysis of fur seal rotavirus. (A) Schematic representation of the genome of rotaviruses using as an example the group C rotavirus (~17.9 kb). The orange bars represent the contigs from Subantarctic fur seal. (B) Neighbor-joining phylogenetic tree based on the alignment of partial amino acid sequences (307 aa) from the RpRd (segment 1) of 19 rotaviruses. Sequences of groups B, G and H were used as outgroup. The rotavirus sequence from Subantarctic fur seal identified in this study is labeled with a black square. The GenBank accession numbers of the viral sequences are shown in parentheses.

Rotaviruses are non-enveloped segmented dsRNA viruses from the *Reoviridae* family. They belong to the *Rotavirus* genus and their genomes contain 11 segments. Based on sequence and serological analyses of the structural protein VP6, there are seven species, also known as groups, of rotaviruses (A-G), and recently a new group H has been proposed [34]. Rotavirus sequences were detected in the Subantarctic fur seal (Fig 8A) with an amino acid identity from 45–69% to group C rotaviruses. The phylogenetic analysis, performed with a partial VP1 sequence, covering 30% of the complete gene, confirmed closer relatedness to group C rotaviruses (Fig 8B). The VP1 gene, which encodes the RdRp, is well conserved within the genus and may be also used for to differentiate rotavirus species [35,36].

### Hepevirus

**Fig 9:**
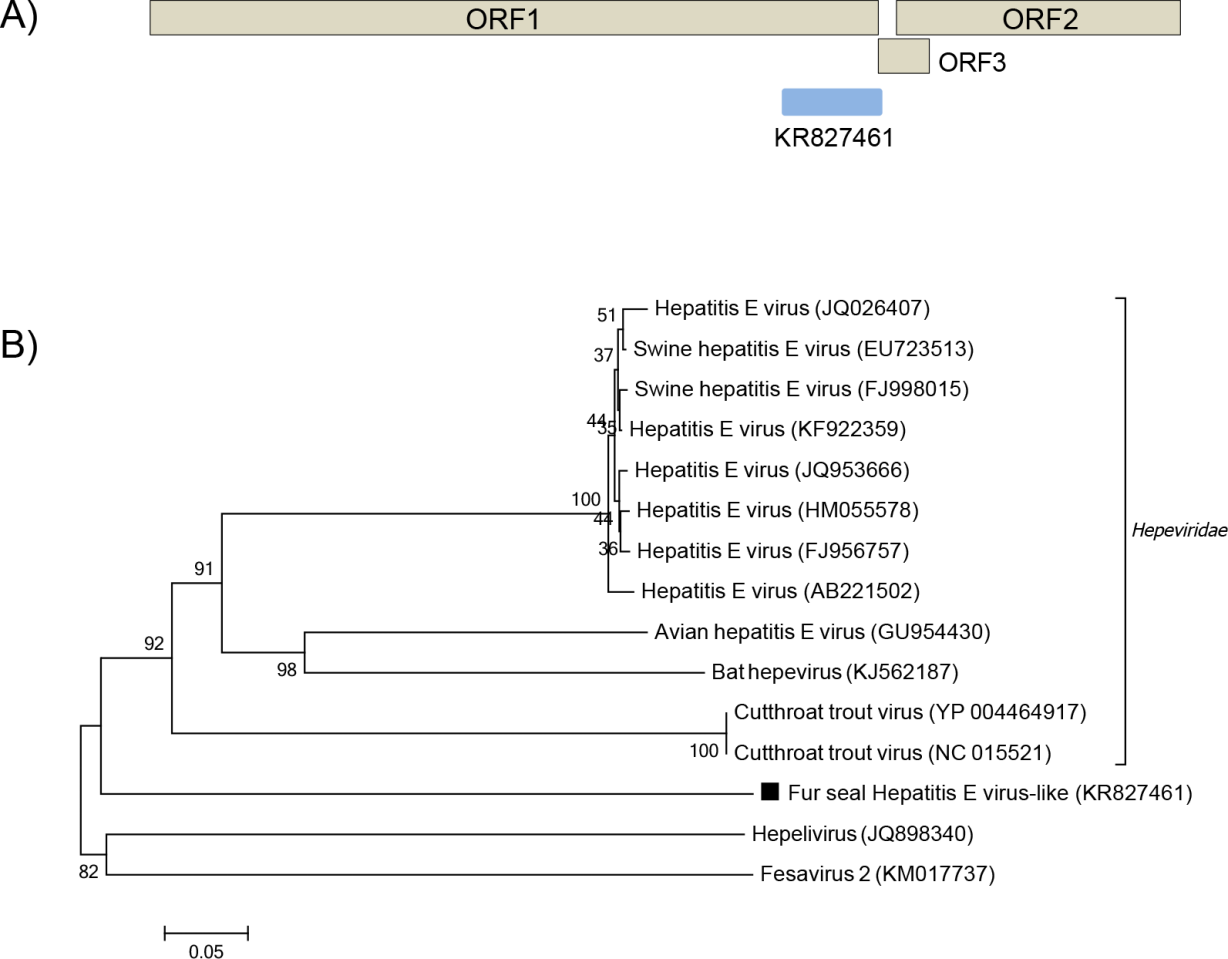
Phylogenetic analysis of fur seal hepevirus-like. (A) Schematic representation of the genome of hepeviruses using as an example the hepatitis E virus (~7.2 kb). The blue bar represents the contig from South American fur seal. (B) Neighbor-joining phylogenetic tree based on the alignment of partial amino acid sequences (182 aa) from the polyprotein of 15 hepeviruses. *Hepelivirus* and *Fesavirus 2* were used as outgroup. The hepevirus-like virus sequence from South American fur seal identified in this study is labeled with a black square. The GenBank accession numbers of the viral sequences are shown in parentheses.

Hepeviruses are non-enveloped, positive sense ssRNA viruses from the *Hepeviridae* family, which is divided in two genera: *Orthohepevirus* and *Piscihepevirus* [37,38]. In this study, a sequence of 661bp with low amino acid identity (27%) to the polyprotein gene of hepeviruses was detected in the South American fur seal (Fig 9A). Phylogenetic analysis of partial sequences of the polyprotein of hepeviruses and hepevirus-like viruses was performed (Fig 9B).

### Sapovirus

**Fig 10:**
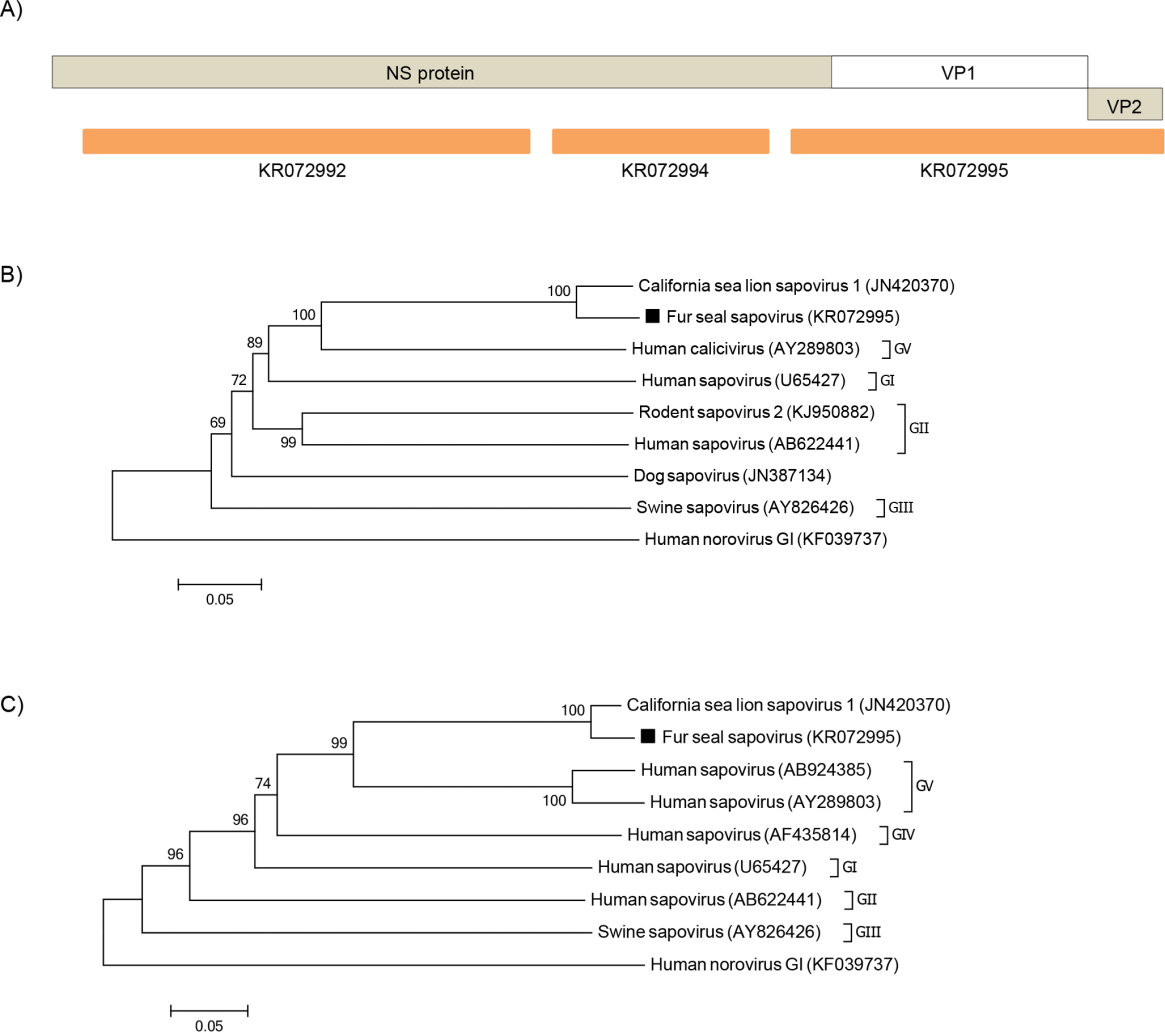
Phylogenetic analysis of fur seal sapovirus. (A) Schematic representation of the genome of sapoviruses using *California sea lion sapovirus 1* (~7.5 kb—JN420370.2) as a reference. The orange bars represent the contigs from Subantarctic fur seal. (B) Neighbor-joining phylogenetic tree based on complete nucleotide sequences from the VP1 gene of 9 caliciviruses. *Human norovirus* was used as outgroup. (C) Neighbor-joining phylogenetic tree based on the alignment of nearly-complete nucleotide sequences from the VP2 gene of 9 caliciviruses. *Human norovirus* was used as outgroup. The sapovirus sequences from Subantarctic fur seal from this study used in phylogenetic analyses are labeled with a black square. The GenBank accession numbers of the viral sequences are shown in parentheses.

The genus *Sapovirus* consists of non-enveloped, positive sense ssRNA viruses of the *Caliciviridae* family. At present, five genogroups have been recognized based on VP1 sequence analyses [39]. Sapovirus sequences were detected in the Subantarctic fur seal samples and results include contigs covering over 90% of the *California sea lion sapovirus 1* (CslSaV1) genome (Fig 10A) while sharing 98% amino acid identity and 89% nucleotide sequence identity. Phylogenetic analysis of complete VP1 gene and nearly complete VP2 are shown in Fig 10B and 10C, respectively.

## Discussion

This study has detected enteric viruses in the fecal samples of two species of fur seals that occur in the coast of Rio Grande do Sul, South of Brazil. Such viruses belong to families whose members are either apathogenic or known to cause disease in mammals. Anelloviruses were detected in both species of fur seals examined. Based on anelloviruses demarcation criteria, ORF1 sequences must have a divergence higher than 56 and 35% for genus and species, respectively [25].The sequences with the highest similarity to seal anelloviruses displayed a 60% divergence, suggesting that we found a new genus of *Anelloviridae*. For the sequences with higher similarity to *Torque teno sus virus 1a*, the divergence was of 26%, which may indicate they belong to the same genus, *Iotatorquevirus*. Anelloviruses, which in most cases are not associated to any particular disease, have been detected in seals and sea lions involved in mortality events and virus-specific seroconversion of seals has been demonstrated, suggesting that such animals are indeed susceptible to a productive infection following natural contact with the virus [11,40–42].

Parvovirus sequences were detected in the two species of fur seals. Members of a same genus within the *Parvoviridae* should share at least 30% amino acid identity in the predicted NS1 sequence, and less than 30% identity when compared to other genera [26]. Although only partial sequences from NS1 were detected in both species, all of them share more than 30% amino acid identity with members of the *Protoparvovirus* genus, which was also shown by phylogenetic analysis, suggesting a new species within the genus. Parvoviruses have been detected in pinnipeds [43,44], and members of different genera, including bocaviruses, dependoviruses and a novel parvovirus named *Sesavirus* have been detected in California sea lions [11,45]. Parvoviruses cause infections that can manifest through a variety of illnesses including leukopenia, myocarditis, gastroenteritis, as well as asymptomatically, and have been detected in healthy and debilitated pinnipeds [44–48]. Parvoviruses are also known to be transmitted between wild and domestic species [49,50].

Picornaviruses, which have been found in marine mammals [51], have also been detected in this study. Here, two distinct picornaviruses were detected: one more similar to HAV and AEV and other similar to FSVA. According to the *Picornaviridae* genus demarcation criteria, different genera should share less than 40%, 40% and 50% amino acid identity in P1, P2 and P3, respectively [30]. Analyses of partial sequences of the polyprotein of the picornavirus similar to HAV and AEV showed that the amino acids identities to members of *Hepatovirus* and *Tremovirus* genera were below these cut-offs. The only exception was one sequence that shared 52% amino acid identity with HAV VP2. A higher identity in this region, however, can be expected within members of *Hepatovirus* and *Tremovirus* according to previous studies [31]. Based on these values, a possible novel picornavirus more closely related to HAV and AEV was detected in both fur seal species.

In addition, a new putative species within the recently recognized genus *Sakobuvirus* was identified in the Subantarctic fur seal, here named *Fur seal sakobuvirus* (FSSV). These share an amino acid identity of at least 50% with *Feline sakobuvirus A* when comparing all polyprotein coding regions, indicating that FSSV belongs to the same genus. To date, FSVA was the sole member of the genus; the genome reported here corresponds to the first description of a sakobuvirus in another animal species, which was first found in cat feces [52].

Other potential novel enteric virus was also identified. A genogroup I picobirnavirus was detected in South American fur seals. This fur seal picorbirnavirus is distinct from the *Otarine picobirnavirus* previously found in California sea lions [53] and may represent a new species within the genus. Picobirnaviruses have been detected both in asymptomatic and symptomatic animals, including humans, and an etiologic association with diarrhea is not fully established. However, coinfections of picobirnaviruses with other enteric viruses are not uncommon, can be opportunistic, and may also have a synergistic effect [54–57].

A rotavirus related to group C was found in Subantarctic fur seals. Group C rotaviruses have been associated with sporadic outbreaks in humans and other animals such as pigs and bovines [58–61]. Other studies have detected rotaviruses in marine mammals: anti-group A rotavirus antibodies were first found in Galapagos sea lions and fur seals [62] and RNA sequences from rotaviruses related to lineage B were also detected in California sea lions [11]. Here, results show that more groups of rotaviruses, other than groups A and B, can circulate in marine mammals.

A hepevirus-like sequence was detected in South American fur seals. Hepeviruses, such as Hepatitis E virus (HEV), have been detected in mammals and birds [37,63]. HEVs can cause asymptomatic infections to acute hepatitis and are known zoonotic agents [64]. Recently, a new member of the *Hepeviridae* family was identified in cutthroat trout, a fish that occurs in the Pacific ocean in North America [65] and unclassified hepevirus-like sequences named hepelivirus and fesavirus-2 were detected in untreated sewage and cat feces, respectively [66,67]. Phylogenetic analysis of the predicted partial polyprotein sequences of the hepevirus-like identified here showed closer relatedness to other members of the *Hepeviridae* family than to the unclassified hepelivirus and fesavirus-2. Although its low amino acid identity (<30%) might indicate a novel member of the *Hepeviridae* family, more parts of the polyprotein would need to be sequenced to better taxonomically allocate it.

A sapovirus was detected in samples from Subantarctic fur seals, which is genetically closely related to CslSaV1 that was previously detected in a California sea lion with severe osteomyelitis and nephrolithiasis [11]. Subantarctic fur seals are found in South Atlantic and Indian oceans and our data shows that a very similar sapovirus circulates among fur seals from the southern hemisphere, in addition to the ones that occur in the northern hemisphere. Caliciviruses have been isolated from marine mammals and are known to cause vesicular lesions and diarrhea in those animals [10,44,68]. Sapoviruses can cause gastroenteritis and have been associated with diarrhea in animals [69–71].

Knowledge on viruses in wildlife is still a barely explored field. Occasionally, viral infections have been associated to epidemics in marine mammals, such as anelloviruses infections, whose pathogenic role remains to be determined [41,42]. Additionally, marine mammals may on occasions be exposed to humans, farm animals or pets, which may represent a risk of cross-species transmission of pathogens and zoonoses [10]. Such risk of transmission to humans, for example, was reported with an avian influenza virus isolated from harbor seals [72]. Besides, it is not uncommon to find dogs in contact with carcasses of these animals found ashore, which may give rise to emerging infectious diseases and transmission of known viruses, as already reported with morbilliviruses [73]. An historical example of cross-species transmission occurred with *San Miguel sea lion virus* that infects marine mammals. This calicivirus is nearly identical to *Vesicular exanthema of swine virus*, eradicated in swine since 1956, and was able to cause an identical disease in pigs fed with infected carcasses of pinnipeds [74]. Sequences of viruses belonging to viral families known to be transmitted between wild, domestic and farm animals were detected in the present study: parvoviruses, hepeviruses and caliciviruses.

Indisputably, factors such as diet, age and different geographical distributions factors could have contributed to the virome profile of both fur seal species [75]. Based on their lengths and weight, most of the fur seals were classified as juveniles—only one Subantarctic fur seal was an adult animal–and one can expect that juvenil animals are more susceptible to viral infections than adults [76]. Fur seals are carnivores and can feed on a variety of preys. Subtantarctic fur seals mostly feed on fish and cephalopods whereas South American fur seals main diet consists of fish and crustaceans [12,77,78]. Their diet can impact on the virome of each fur seal species and could explain, for example, the detection of eukaryotic viruses that do not infect mammals. The use of fecal samples can allow the detection of sequences that may be originated from different hosts rather than the fur seals. Rotaviruses and sapoviruses were only detected in Subantarctic fur seals whereas picobirnaviruses and a hepevirus-like were only found in South American fur seals. Sequences of anelloviruses, parvoviruses and picornaviruses were detected in both species of fur seals. These have also been reported in seals from the northern hemisphere, indicating the widespread distributions of viruses of such families in pinnipeds [11,41,42,51]. Furthermore, the occurrence of a very genetically closely related sapovirus that infects California sea lions in Subantarctic fur seals shows that viruses previously isolated in the North can also circulate in the South, infecting pinniped populations over a large geographical range.

Although the nucleotide sequences reported in this study do not comprise full genomes, this initial characterization contributes to the knowledge of the viral populations that occur in fur seals, and has identified potential novel viruses that may be of interest for future studies. This is the first study to use next generation sequencing to explore the viral diversity of southern hemisphere marine mammals. The findings presented here are expected to help to understand how viral infections in pinnipeds may impact the health of the pinniped population and its potential as sources of viruses which may potentially infect other animal species.
